# Dendritic Properties Control Energy Efficiency of Action Potentials in Cortical Pyramidal Cells

**DOI:** 10.3389/fncel.2017.00265

**Published:** 2017-09-01

**Authors:** Guosheng Yi, Jiang Wang, Xile Wei, Bin Deng

**Affiliations:** School of Electrical and Information Engineering, Tianjin University Tianjin, China

**Keywords:** dendrite, action potential, two-compartment model, Na^+^ entry, metabolic efficiency

## Abstract

Neural computation is performed by transforming input signals into sequences of action potentials (APs), which is metabolically expensive and limited by the energy available to the brain. The metabolic efficiency of single AP has important consequences for the computational power of the cell, which is determined by its biophysical properties and morphologies. Here we adopt biophysically-based two-compartment models to investigate how dendrites affect energy efficiency of APs in cortical pyramidal neurons. We measure the Na^+^ entry during the spike and examine how it is efficiently used for generating AP depolarization. We show that increasing the proportion of dendritic area or coupling conductance between two chambers decreases Na^+^ entry efficiency of somatic AP. Activating inward Ca^2+^ current in dendrites results in dendritic spike, which increases AP efficiency. Activating Ca^2+^-activated outward K^+^ current in dendrites, however, decreases Na^+^ entry efficiency. We demonstrate that the active and passive dendrites take effects by altering the overlap between Na^+^ influx and internal current flowing from soma to dendrite. We explain a fundamental link between dendritic properties and AP efficiency, which is essential to interpret how neural computation consumes metabolic energy and how biophysics and morphologies contribute to such consumption.

## Introduction

Cortical pyramidal cells have powerful abilities to process incoming signals, which are metabolically expensive. They adopt diverse patterns of APs to encode information and perform computation. This is a primary process that consumes energy within a pyramidal cell (Sengupta et al., 2010; Niven, 2016). The availability of energy supply in the brain not only limits the survival (Diaz et al., 2012), development (Schuchmann et al., 2005), cellular maintenance (Engl and Attwell, 2015), and evolution (Niven and Laughlin, 2008) of a neuron, which also constrains its computational power (Attwell and Laughlin, 2001; Attwell and Gibb, 2005; Alle et al., 2009; Sengupta et al., 2010; Yu et al., 2012; Niven, 2016). Importantly, energy efficiency plays a critical role in coding schemes and strategies of neural system, which can determine information rate, coding capacity, signal transfer manner and transmission reliability (Schreiber et al., 2002; Sengupta et al., 2013, 2014; Yu and Liu, 2014; Wang et al., 2015; Zhang et al., 2015; Niven, 2016; Yu et al., 2016). In particular, there are trade-offs between energy expenditure and information processing (Schreiber et al., 2002; Sengupta et al., 2013; Yu and Liu, 2014; Niven, 2016). Such special relationship between them can govern the numbers and types of signaling molecules, synapses and ion channels used by cells, which also strongly influences neuronal anatomy and physiology at different scales (Schreiber et al., 2002; Niven, 2016). Investigating how metabolic energy is efficiently used to generate APs in pyramidal cells is therefore essential for deeply understanding their computation. It is also significant for interpreting neural circuit functions and functional imaging signals related to metabolic mechanisms (Niven and Laughlin, 2008; Sengupta et al., 2010; Yu et al., 2012; Niven, 2016).

In cortical pyramidal cells, the AP is initiated in the axon initial segment (AIS; Stuart et al., 1997), which arises from the non-linear interaction between inward and outward currents (Prescott et al., 2008). Two common ions include Na^+^ and K^+^. According to their concentration gradients, Na^+^ flows into the cell and K^+^ out of the cell. When membrane depolarization reaches a threshold level (i.e., AP threshold), inward Na^+^ current becomes self-sustaining and vast number of Na^+^ ions flow into the cell. It effectively depolarizes membrane potential and results in the fast upstroke of AP. Strong depolarization activates K^+^ current and lets K^+^ ions exit the cell. The efflux of K^+^ hyperpolarizes membrane potential, which generates the falling phase of AP. To re-establish ion gradients and maintain signaling, the Na^+^/K^+^ pump extrudes Na^+^ ions and imports K^+^ ions during each AP (Kandel et al., 2012; Niven, 2016). This subcellular process is performed by consuming significant quantities of metabolic energy provided by the hydrolysis of ATP molecules (Hasenstaub et al., 2010; Sengupta et al., 2010; Howarth et al., 2012; Niven, 2016). The Na^+^/K^+^ pump hydrolyses one ATP when it imports two K^+^ ions to the cell and extrudes three Na^+^ ions out of the cell.

The metabolic energy consumed by an AP is tightly related to the entry of Na^+^ ions into the cell (Carter and Bean, 2009; Yu et al., 2012). If Na^+^ influx is confined to the rising phase of the AP and K^+^ efflux to its falling phase, there would be perfect energy efficiency. However, the kinetics of voltage-gated channels causes the overlap between Na^+^ and K^+^ currents (Crotty et al., 2006; Carter and Bean, 2009; Hasenstaub et al., 2010). Such overlap would merely result in an electrically neutral exchange of positive ions, which makes Na^+^ influx less efficient in generating membrane depolarization, thus inflating energy cost. The complete separation of opposite currents decreases energy expenditure to close to the minimum possible, which increases Na^+^ entry efficiency. The extent of the overlap between opposite currents determines the efficiency of both Na^+^ entry and metabolic energy, which is highly variable among neurons. It is shown that channel types, densities and kinetics (Crotty et al., 2006; Crotty and Levy, 2007; Hasenstaub et al., 2010; Sengupta et al., 2010; Moujahid and d'Anjou, 2012; Yi et al., 2016a), AP shape (Carter and Bean, 2009), spike threshold dynamics (Yi et al., 2015a), and temperature (Moujahid and d'Anjou, 2012; Yu et al., 2012) could all influence Na^+^ entry efficiency through altering the overlap of opposite currents. To interpret the energy cost in pyramidal cells, it is essential to understand the efficiency of Na^+^ entry during an AP and its relationship with biophysical properties and morphology of the cell.

Dendrites are the primary sites for receiving signals in cortical pyramidal cells (Spruston, 2008), which also deliver them to the initiation zone of APs. Their tree-like structure disperses input sites and effectively extends the receptive field of the cell (Spruston, 2008; Grienberger et al., 2015). Particularly, each dendritic branch is an independent processing and signaling unit (Branco and Häusser, 2010), which can perform local computation by integrating synaptic input. Such local integration is critically dependent on their passive and active properties (Spruston, 2008; Stuart and Spruston, 2015; Tran-Van-Minh et al., 2015). Specifically, the passive properties, such as axial resistance, diameter, branching, and the distance of the synapse from the soma, mainly filter and attenuate synaptic inputs as they spread to the soma, resulting in passive/sublinear integration (Stuart and Spruston, 2015; Tran-Van-Minh et al., 2015). The active currents can either boost or attenuate dendritic depolarization. Activating the active conductance of inward currents initiates dendritic spike. Such local threshold-dependent regenerative response boosts inputs, resulting in active/supralinear integration (Major et al., 2013; Tran-Van-Minh et al., 2015). On the contrary, activating outward K^+^ current produces sublinear integration (Hu et al., 2010; Tran-Van-Minh et al., 2015). The outcome of dendritic integration directly participates in AP initiation and alters the final output. Such non-linear transformation conferred by the dendrites can effectively increase the computational ability of pyramidal neurons (Spruston, 2008; Branco and Häusser, 2010; Major et al., 2013; Grienberger et al., 2015; Stuart and Spruston, 2015; Tran-Van-Minh et al., 2015). However, it is still unknown how dendrites influence the metabolic efficiency of somatic/axonal APs, leaving important questions unanswered. What roles do passive and active properties play in the efficiency of Na^+^ entry? Do they affect AP efficiency also through altering the overlap of Na^+^ and K^+^ currents in the soma/axon? What properties of the dendrites are contributory factors for producing an energy efficient AP?

Here we attempt to answer these questions by numerical simulations of biophysically-based models. We develop three two-compartment models to describe passive and active dendrites. The excess Na^+^ entry ratio (Carter and Bean, 2009; Yu et al., 2012) is applied to quantify how efficiently Na^+^ influx is used for AP depolarization. By relating dendritic properties to the internal current flowing from the soma to the dendrite, and by identifying how such current overlaps with Na^+^ influx in the soma, we explain how passive and active dendrites participate in the energy efficiency of APs in cortical pyramidal cells.

## Methods

The biophysically-based two-compartment models are used in our simulations. Such type of model is the minimal structure to capture the interactions between dendrites and soma/axon in cortical pyramidal cells. One chamber represents apical dendrites, and the other one describes the soma plus the AIS. APs are initiated and recorded in latter chamber. We develop three models to quantify how the passive and active properties of the dendrites affect the energy efficiency of APs.

### Model I

Our starting model is derived from the Pinsky-Rinzel (PR) model (Pinsky and Rinzel, 1994). We block active currents in its dendritic chamber, and create a simple model with passive dendrite, i.e., model I. To generate APs, we include inward Na^+^ and outward K^+^ currents in somatic chamber. The current-balance equations for model I are described by

(1) $$\begin{array}{l} {\text{C}}_{\text{m}}\frac{\text{d}V_{\text{S}}}{\text{d}t}=-\frac{g_{\text{c}}(V_{\text{S}}-V_{\text{D}})}{p}-I_{\text{Na}}-I_{\text{K}}-I_{\text{SL}}\\ {\text{C}}_{\text{m}}\frac{\text{d}V_{\text{D}}}{\text{d}t}=I_{\text{D}}+\frac{g_{\text{c}}(V_{\text{S}}-V_{\text{D}})}{1-p}-I_{\text{DL}} \end{array}$$

where *V*_S_ and *V*_D_ are the transmembrane potentials of soma and dendrite. *I*_D_ (in μA/cm^2^) is the input current applied to activate neurons. ${\text{C}}_{\text{m}}=1\;\mu \text{F}/{\text{cm}}^{2}$ is the membrane capacitance. *p* and 1 − *p* are two morphological parameters, which respectively describe the proportion of cell area taken up by the soma and the dendrite. Two compartments are separated by an internal coupling conductance *g*_c_ (in mS/cm^2^). *I*_SD_ = *g*_c_(*V*_S_ − *V*_D_)/*p* is the internal current flowing from the soma to the dendrite along internal conductance. Other ionic currents included in model I are

(2) $$\begin{array}{l} I_{\text{Na}}={\bar{\text{g}}}_{\text{Na}}m_{\infty }^{3}(V_{\text{S}})h(V_{\text{S}}-{\text{E}}_{\text{Na}})\\ I_{\text{K}}={\bar{\text{g}}}_{\text{K}}n^{4}(V_{\text{S}}-{\text{E}}_{\text{K}})\\ I_{\text{SL}}={\text{g}}_{\text{SL}}(V_{\text{S}}-{\text{E}}_{\text{SL}})\\ I_{\text{DL}}={\text{g}}_{\text{DL}}(V_{\text{D}}-{\text{E}}_{\text{DL}}) \end{array}$$

The gating variables, including activation and inactivation variables for inward *I*_Na_ (i.e., *m* and *h*) and activation variable for outward *I*_K_ (i.e., *n*), satisfy following first-order kinetics

(3) $$\frac{\text{d}x}{\text{d}t}=\alpha_{x}(V_{\text{S}})(1-x)-\beta_{x}(V_{\text{S}})x$$

where *x* ∈ {*m, h, n*}. Here the details of each current follow the descriptions by Wang (1998). For each gating variable, the transition rate α_*x*_ and β_*x*_ are

(4) $$\begin{array}{l} \alpha_{m}=-0.1(V_{\text{S}}+33)/\left\{\exp[-0.1(V_{\text{S}}+33)]-1\right\}\;\\ \beta_{m}=4\exp[-(V_{\text{S}}+58)/12\;]\\ \alpha_{h}=0.07\exp[-(V_{\text{S}}+50)/10\;]\\ \beta_{h}=1/\left\{\exp[-0.1(V_{\text{S}}+20)]+1\right\}\\ \alpha_{n}=-0.01(V_{\text{S}}+34)/\left\{\exp[-0.1(V_{\text{S}}+34)]-1\right\}\\ \beta_{n}=0.125\exp[-(V_{\text{S}}+44)/25\;] \end{array}$$

In model I, the activation variable *m* of *I*_Na_ is replaced by its steady state *m*_∞_ = α_*m*_/(α_*m*_ + β_*m*_) (Wang, 1998). ${\bar{\text{g}}}_{\text{Na}}=45\text{mS}/\text{c}{\text{m}}^{\text{2}},$ ${\bar{\text{g}}}_{\text{K}}=18\;\text{mS}/\text{c}{\text{m}}^{\text{2}},$ ${\text{g}}_{\text{SL}}=0.1\;\text{mS}/\text{c}{\text{m}}^{\text{2}}$, and ${\text{g}}_{\text{DL}}=0.1\text{mS}/\text{c}{\text{m}}^{\text{2}}$ are the maximum conductances associated with the currents. E_Na_ = 55mV, E_K_ = −80mV, E_SL_ = −65mV, and E_DL_ = −65mV are the reversal potentials for relevant channels. This model is used to simulate the effects of varying morphological parameter *p* and coupling conductance *g*_c_ on the energy efficiency of APs, i.e., the passive properties of dendritic chamber.

### Model II

To determine how active dendrites affect AP efficiency, we introduce an inward Ca^2+^ current *I*_Ca_ into the dendritic chamber of model I, and develop another two-compartment model, i.e., model II. The Ca^2+^ current is given by (Mainen and Sejnowski, 1996)

(5) $$I_{\text{Ca}}={\bar{\text{g}}}_{\text{Ca}}s^{2}c(V_{\text{D}}-{\text{E}}_{\text{Ca}})$$

Here ${\bar{\text{g}}}_{\text{Ca}}=0.8\text{mS}/\text{c}{\text{m}}^{\text{2}}$ and E_Ca_ = 140mV, which are modified from the PR like models (Pinsky and Rinzel, 1994; Wang, 1998; Park et al., 2005). Kinetics of activation variable *s* and inactivation variable *c* obeys

(6) $$\begin{array}{l} \frac{\text{d}s}{\text{d}t}=\frac{s_{\infty }(V_{\text{D}})-s}{\tau_{s}(V_{\text{D}})}=\alpha_{s}(V_{\text{D}})(1-s)-\beta_{s}(V_{\text{D}})s\\ \frac{\text{d}c}{\text{d}t}=\frac{c_{\infty }(V_{\text{D}})-c}{\tau_{c}(V_{\text{D}})}=\alpha_{c}(V_{\text{D}})(1-c)-\beta_{c}(V_{\text{D}})c \end{array}$$

The transition rate for gating variable *s* and *c* are

(7) $$\begin{array}{l} \alpha_{s}=0.005(V_{\text{D}}+27)/\left\{1-\exp[-(27+V_{\text{D}})/3.8\;]\right\}\\ \beta_{s}=0.94\exp[-(V_{\text{D}}+75)/17\;]\\ \alpha_{c}=0.000457\exp[-(V_{\text{D}}+13)/50\;]\\ \beta_{c}=0.0065/\left\{1+\exp[-(V_{\text{D}}+15)/28\;]\right\}\; \end{array}$$

The kinetics of gating variable *s* and *c* is the same as that described by Mainen and Sejnowski (1996). This model is used to examine how activating Ca^2+^ current in dendritic chamber affects AP efficiency.

### Model III

By introducing an outward current *I*_KAHP_ into the active dendrite of model II, we derive model III. *I*_KAHP_ is a voltage-independent, Ca^2+^-activated K^+^ current, and activating it causes spike-frequency adaptation (SFA) on slow timescales. This inhibitory current is described by (Pinsky and Rinzel, 1994; Park et al., 2005)

(8) $$I_{\text{KAHP}}={\bar{\text{g}}}_{\text{KAHP}}\;q(V_{\text{D}}-{\text{E}}_{\text{K}})$$

Here the maximal conductance is ${\bar{\text{g}}}_{\text{KAHP}}=5\text{mS}/\text{c}{\text{m}}^{\text{2}}$. Kinetics of activation variable *q* is given by

(9) $$\frac{\text{d}q}{\text{d}t}=\frac{q_{\infty }(V_{\text{D}})-q}{\tau_{q}}$$

where time constant is τ_*q*_ = 800ms, and steady-state function is *q*_∞_ = α_*q*_/(α_*q*_ + β_*q*_). The transition rates for *I*_KAHP_ are α_*q*_ = min(0.00002[Ca], 0.01) and β_*q*_ = 0.001. [Ca] is the intracellular Ca^2+^ concentration, and its kinetics follows

(10) $$\frac{\text{d}[\text{Ca}]}{\text{d}t}=-0.13I_{\text{Ca}}-0.075[\text{Ca}]$$

This model is used to simulate the effects of activating hyperpolarizing current in dendritic chamber on AP efficiency.

We apply excess Na^+^ entry ratio to quantify the efficiency of Na^+^ entry during an AP. Following Carter and Bean (2009), it is defined as the ratio of total actual Na^+^ entry Q_total_ during the AP to the minimal Na^+^ load Q_min_ necessary for producing the voltage change of the AP. For a spike train recorded in our simulations, an AP is required to begin and end below or closest to the resting potential and cross at least 0 mV at maximum (Hasenstaub et al., 2010). In this case, all of the active ionic currents take place during the defined interval of the AP (Crotty et al., 2006). Total Na^+^ load Q_total_ per spike is calculated by integrating the Na^+^ current curve over the duration of the AP (Carter and Bean, 2009; Hasenstaub et al., 2010; Sengupta et al., 2010, 2013; Yu et al., 2012), i.e., Q_total_ = ∫ *I*_Na_(*t*)d*t*. Minimum charge Q_min_ necessary to produce the depolarization of the AP is calculated as Q_min_ = C_m_Δ*V*_S_, where C_m_ is the membrane capacitance. As mentioned in Introduction, an AP is initiated when enough membrane depolarization accumulates to bring *V*_S_ to reach spike threshold. After that, inward Na^+^ current becomes self-sustaining to result in a positive feedback loop and generate the rising phase of the AP. To calculate the minimum charge Q_min_, we measure Δ*V*_S_ as the change of somatic voltage from spike threshold (d*V*_S_/d*t* = 20mV/ms) to the peak of the AP (d*V*_S_/d*t* = 0mV/ms) (Carter and Bean, 2009; Yu et al., 2012; Ju et al., 2016). The excess Na^+^ entry ratio calculated in this way has been widely applied to describe the metabolic efficiency of the APs in different kinds of cells (Attwell and Laughlin, 2001; Alle et al., 2009; Carter and Bean, 2009; Sengupta et al., 2010; Moujahid and d'Anjou, 2012; Yu et al., 2012; Niven, 2016; Yi et al., 2016a). It is shown that a lower value means most of Na^+^ entry is confined to the depolarizing phase of the spike, and there are less overlaps between inward and outward currents, corresponding to a relatively efficient AP. On the contrary, a higher value of Na^+^ entry ratio indicates that more of metabolic energy is devoted to the reversal of ion exchanges, which corresponds to an inefficient AP. The temporal overlap of inward *I*_Na_ and outward *I*_K_ (i.e., Q_overlap_) is measured as the difference between the total Na^+^ load during an AP and the associated depolarizing component of the Na^+^ load (Crotty et al., 2006; Sengupta et al., 2010; Moujahid and d'Anjou, 2012).

Earlier studies (Crotty et al., 2006; Hasenstaub et al., 2010; Howarth et al., 2012; Sengupta et al., 2013) determine the energy cost of an AP by the amount of ATP molecules expended in the spike duration. It is known that the Na^+^/K^+^-ATPase hydrolyses one ATP per three Na^+^ extruded and two K^+^ imported. Based on this fact, they measure the amount of Na^+^ (or K^+^) ions consumed in the AP. The total Na^+^ (or K^+^) load is then converted to the number of ATP molecules by using the 3:1 (or 2:1) stoichiometry of the Na^+^/K^+^-ATPase. Thus, there is a direct relationship between total Na^+^ load Q_total_ during an AP and its energy cost. Following Sengupta et al. (2010), we use Q_total_ to define the energy consumption of an AP in our simulations. Note that such definition of AP cost is not accurate, but it does not alter our predictions about how dendrites affect the metabolic efficiency of somatic APs.

The shape of the simulated APs is characterized by their height and half-width. The height is determined by measuring the difference in somatic voltage *V*_S_ from the peak to the most negative voltage reached after the AP (Carter and Bean, 2009; Sengupta et al., 2010; Yu et al., 2012). The half-width is measured as the spike width at half the AP height.

All simulations of the two-compartment models are performed in MATLAB environment. The aforementioned dynamical equations are integrated numerically by using ode23 solver, with a time resolution of 0.001 ms. The computer code for model simulations in present study will be available for public download under the ModelDB section of the Senselab database (https://senselab.med.yale.edu/modeldb/enterCode.cshtml?model=230329).

## Results

### Increasing the proportion of dendritic area decreases Na^+^ entry efficiency of APs

**Figure 1:**
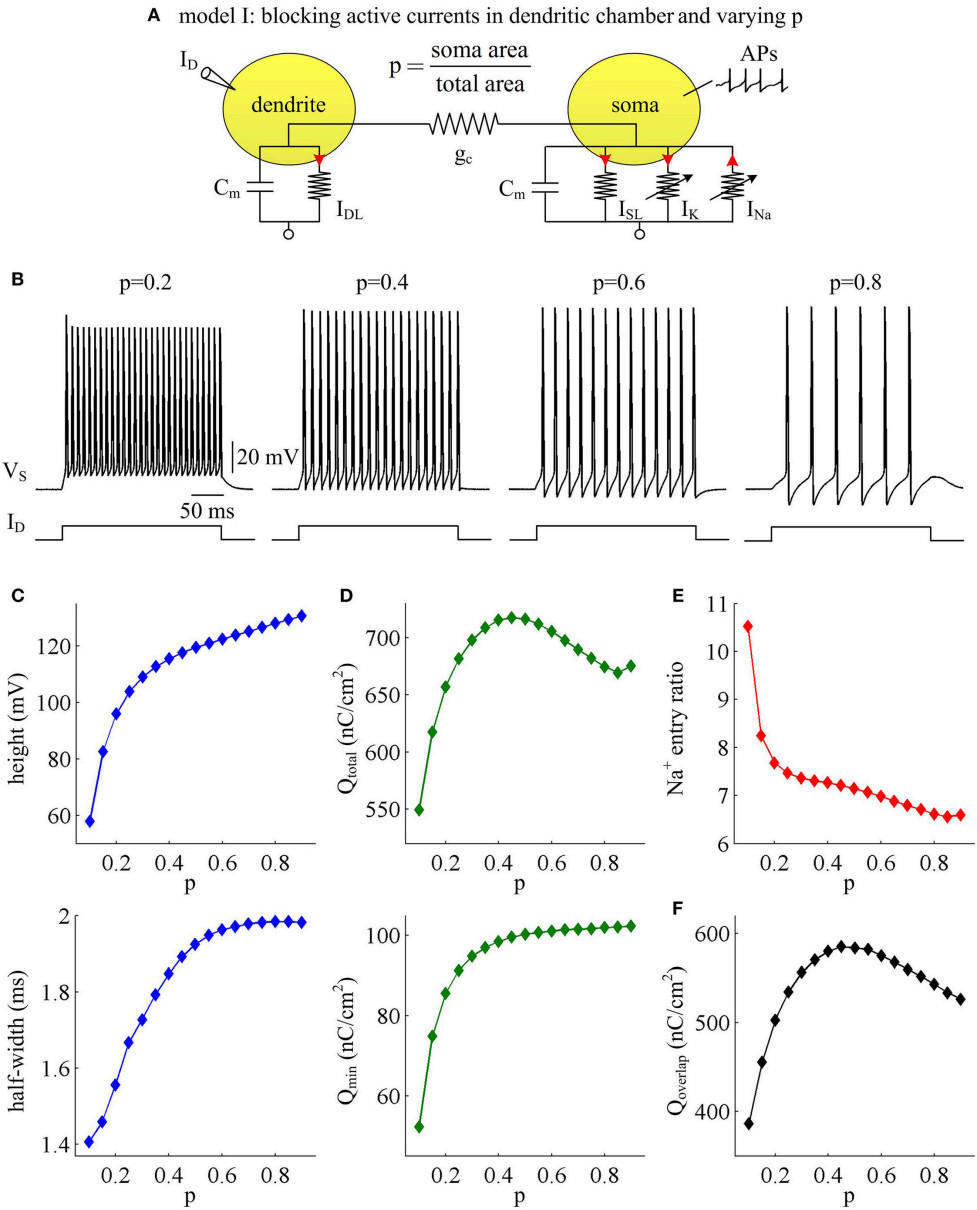
AP shape and metabolic efficiency vary with dendrite area in model I. **(A)** Schematic of model I. There are no active channels in its dendritic chamber. Red arrows indicate the direction of ionic current. We set coupling conductance to $g_{\text{c}}=0.5\;\text{mS}/\text{c}{\text{m}}^{\text{2}}$ and vary morphological parameter *p*. **(B)** Responses of membrane voltage *V*_S_ recorded from the soma with different values of *p*, which are indicated on the top of each panel. **(C)** Both height and half-width of the AP increase as a function of *p*. **(D)** Top panel: total Na^+^ load Q_total_ during an AP is plotted as a function of *p*. Note that Q_total_ reaches a maximum with moderate value of *p*. Bottom panel: minimal Na^+^ load Q_min_ increases as a function of *p*. **(E)** Na^+^ entry ratio decreases as a function of *p*. **(F)** Overlap Na^+^ load Q_overlap_ during an AP is plotted as a function of *p*. Similar to Q_total_, Q_overlap_ also reaches a maximum with moderate value of *p*. Dendritic input is $I_{\text{D}}=3\;\mu \text{A}/\text{c}{\text{m}}^{\text{2}}$.

Our first step is to examine the effects of passive properties of the dendrites. A simple two-compartment model is adopted to simulate APs generated in the soma of cortical pyramidal cells, i.e., model I (Figure 1A). There are no active currents in its dendritic chamber. This model includes a morphological parameter *p* that describes the proportion of soma area, and we use it to characterize dendritic geometry. In our two-compartment models, increasing *p* means there is a decrease in dendrite area. We apply constant input *I*_D_ to activate model I and simultaneously record APs generated in somatic chamber. As parameter *p* varies, the spike trains are always periodic (Figure 1B), but the AP shape alters. Specifically, the height and half-width of the AP both increase with parameter *p* (Figure 1C), i.e., decreases with dendrite area.

We adopt excess Na^+^ entry ratio to quantify the metabolic efficiency of the recorded APs, and examine how dendrite area affects this quantity. It is shown that the total Na^+^ load Q_total_ during an AP increases at first and then decreases with parameter *p* (Figure 1D, top). This means that the metabolic cost per AP is low with large dendrite area, i.e., energy efficient. The APs with moderate size of dendritic chamber are metabolically inefficient, since they have high energy cost. Unlike Q_total_, the minimal Na^+^ load Q_min_ needed to generate the upstroke of the AP increases monotonically in the observed range of *p* (Figure 1D, bottom). By calculating Q_total_/Q_min_, we find that the excess Na^+^ entry ratio decreases with parameter *p* (Figure 1E). That is, increasing dendrite area in model I neuron increases its Na^+^ entry ratio and facilitates to reduce AP efficiency. With large dendrite area (i.e., small *p*), the high Na^+^ entry ratio shows that Na^+^ influx is inefficiently used for the depolarization of relevant AP. Here more of the Na^+^ influx during an AP is devoted to the reversal of ion exchanges. These simulations indicate that a small Na^+^ entry ratio does not correspond to low energy cost for individual spikes. The metabolically efficient APs with large dendritic chamber may arise from other factors. We also calculate the temporal overlap Q_overlap_ between Na^+^ and K^+^ currents during the repolarizing component of the AP, which has been shown to be a determinant of metabolic efficiency. Unfortunately, we fail to identify a relationship between overlap load and Na^+^ entry ratio. We find that the overlap of inward Na^+^ and outward K^+^ currents during an AP reaches a maximum with moderate values of *p* (Figure 1F). Therefore, we predict that increasing dendrite area may alter other outward currents to overlap with Na^+^ influx during the APs to result in inefficient use of Na^+^ entry.

**Figure 2:**
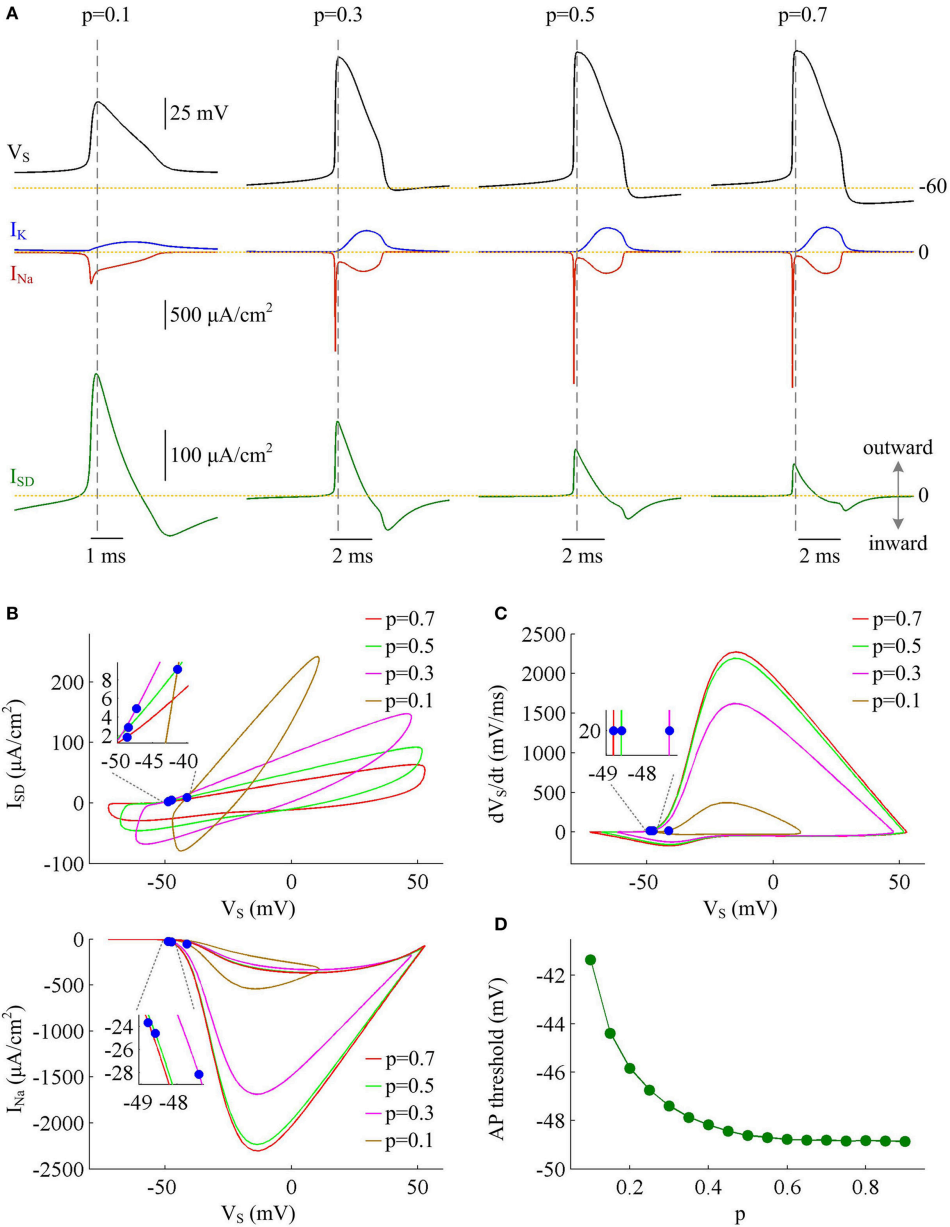
Inefficient Na^+^ entry arises from increased overlap between *I*_SD_ and *I*_Na_ as dendrite area increases. **(A)** *I*_Na_ (red), *I*_K_ (blue), and *I*_SD_ (green) underlying the APs with different values of parameter *p*, which have been indicated on the top of the panels. **(B)** Top panel depicts the relationship between internal current *I*_SD_ and somatic voltage *V*_S_ (i.e., *I*_SD_-*V*_S_ curve) for each AP shown in **(A)**. Bottom panel shows relevant *I*_Na_-*V*_S_ curves. **(C)** Phase plot of d*V*_S_/d*t* vs. *V*_S_ for the APs. Blue dots in **(B,C)** indicate where the APs are initiated. **(D)** AP threshold decreases as a function of *p*. The threshold voltage is defined as the *V*_S_ at which d*V*_S_/d*t* crosses 20 mV/ms during the upstroke of the spike. $I_{\text{D}}=3\;\mu \text{A}/\text{c}{\text{m}}^{\text{2}}$, $g_{\text{c}}=0.5\;\text{mS}/\text{c}{\text{m}}^{\text{2}}$.

To determine how dendrite area affects AP efficiency, we examine the ionic currents underlying the recorded APs in model I neuron. With low morphological parameter *p*, the soma is much smaller than the dendrite. Accordingly, the intensities of *I*_Na_ and *I*_K_ are both relatively weak during an individual spike (Figure 2A, *p* = 0.1). This results in the APs with small height and short half-width (Figures 1B,C), which effectively decreases the metabolic cost of relevant AP. In this case, the small shape of the AP is the primary factor for its high energy efficiency. As parameter *p* gets larger, soma area is increased, which reduces the constraint on ionic channels used for signaling. Then, the activation level of active currents gets higher, especially for inward *I*_Na_. This effectively increases AP size and relevant total Na^+^ load, thus increasing its metabolic cost. Once *p* exceeds 0.5, the soma dominates cell excitability, and there are little changes in either *I*_Na_ or *I*_K_. Unlike two active channels, the internal current *I*_SD_ flowing out of the soma shows a marked decrease within the whole range of *p* (Figure 2A, bottom). The presence of outward *I*_SD_ results in an overlap between with Na^+^ influx during the depolarizing phase of the AP. Such temporal overlap is in effect similar to the overlap between *I*_Na_ and *I*_K_ during the falling phase, which increases total Na^+^ charge required for membrane depolarization. Decreasing the intensity of such inhibitory current effectively reduces Na^+^ load. That is why total Na^+^ load during an AP shows slight decrease once *p* exceeds 0.5. With high values of *p*, there is less outward current prior to AP initiation (Figure 2B, top), and inward Na^+^ faces less competition as it depolarizes membrane potential *V*_S_. Then, *I*_Na_ is able to become self-sustaining at a more hyperpolarized voltage (Figure 2B, bottom), and results in a lower AP threshold (Figures 2C,D). Combined with increased AP peak, the minimal Na^+^ load needed to produce the voltage change of the spike increases with parameter *p*. Further, the overlap between Na^+^ influx and outward *I*_SD_ during the depolarizing phase of an AP decreases as *p* is increased. This makes APs become more effective to use Na^+^ influx to achieve its depolarization, which results in a lower excess Na^+^ entry ratio. These simulations indicate that increasing dendrite area increases the outward level of internal current *I*_SD_, which results in significant overlap between with Na^+^ influx and then decreases the efficiency of Na^+^ entry during an AP.

### Increasing coupling conductance between two compartments decreases AP efficiency

**Figure 3:**
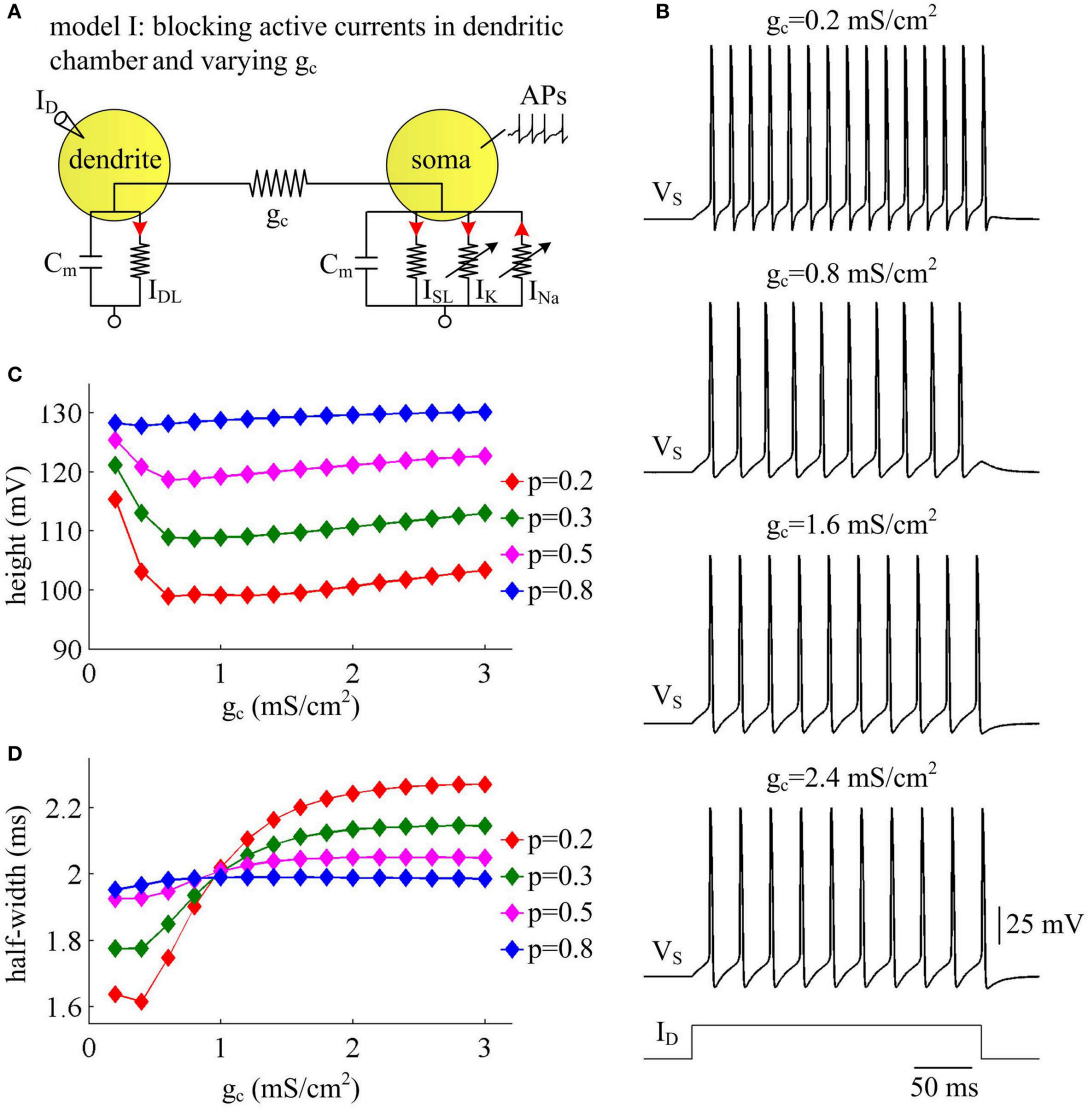
AP shape varies with coupling conductance between chambers in model I. **(A)** We vary coupling conductance *g*_c_ in model I with passive dendrite. **(B)** Sample responses recorded in the soma with different values of *g*_c_, which are indicated on the top of each panel. Data are shown for *p* = 0.5. **(C)** The height of the AP is plotted as a function of *g*_c_ for different values of *p*, which are 0.2, 0.3, 0.5, and 0.8. **(D)** The half-width of the AP is plotted as a function of *g*_c_ for each value of *p*. Dendritic input is $I_{\text{D}}=2\;\mu \text{A}/\text{c}{\text{m}}^{\text{2}}$. Note that the height and half-width for individual spike in **(C,D)** are different from those with same values of *p* and *g*_c_ in Figure 1C. This arises from that stimulus input *I*_D_ used in the case of varying dendrite area is 3 μA/cm^2^. Such higher current injection directly controls the excitability of two-compartment models and alters the shape of output APs.

Coupling conductance *g*_c_ between two compartments is another passive property that controls internal current *I*_SD_ in two-compartment model. In this section, we adopt model I to simulate the effects of varying *g*_c_ on the metabolic efficiency of APs (Figure 3A). In the observed range of *g*_c_, model I neuron generates periodic spike trains to constant input *I*_D_ (Figure 3B). The AP shape shows similar evolutions with *g*_c_ for different values of *p* (Figures 3C,D). Note that the height and half-width of the AP is almost independent of the coupling conductance *g*_c_ with small dendrite area, such as *p* = 0.8. In this case, the soma is much larger than the passive dendrite and dominates the output APs of model I. Thus, increasing *g*_c_ connecting two chambers has little effects on AP shape.

**Figure 4:**
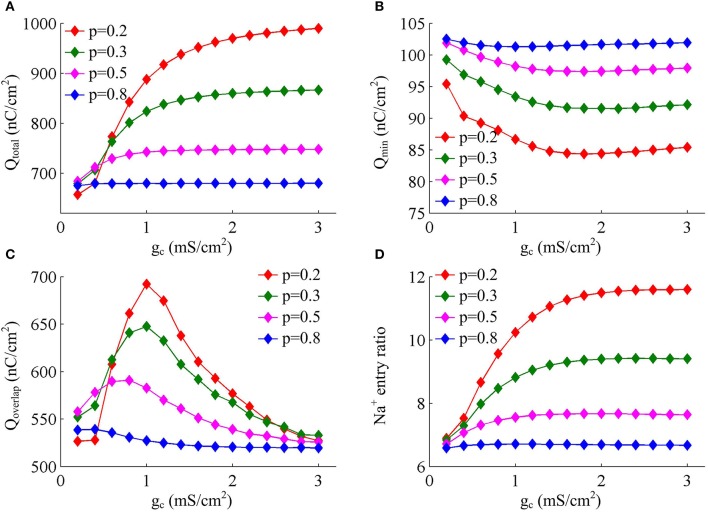
Increasing coupling conductance between chambers reduces AP efficiency in model I. **(A)** Total Na^+^ load Q_total_, **(B)** minimal Na^+^ load Q_min_, **(C)** overlap Na^+^ load Q_overlap_, and **(D)** Na^+^ entry ratio are respectively plotted as a function of *g*_c_ in model I. The value of *p* is 0.2, 0.3, 0.5, and 0.8. Dendritic input is 2μA/cm^2^. Since applied *I*_D_ alters the final output of model I neuron, the items for an AP examined here are different from those with same *p* and *g*_c_ in Figures 1D–F.

Figure 4 shows the total Na^+^ load, the minimal Na^+^ load, the overlap load between Na^+^ and K^+^ currents, and the Na^+^ entry ratio during an AP as *g*_c_ varies. With different values of *p*, these items show similar trends in the observed range of *g*_c_. As coupling conductance increases, the total Na^+^ load during an AP is increased (Figure 4A) while the minimal Na^+^ load is reduced (Figure 4B). Their ratio, i.e., excess Na^+^ entry ratio, is monotonically increased with *g*_c_ (Figure 4D), and relevant AP becomes less efficient to use Na^+^ influx. This indicates that the generation of individual spike requires more energy and becomes metabolically inefficient with high coupling conductance. Once *g*_c_ exceeds 2 mS/cm^2^, total Na^+^ load, minimal Na^+^ load and Na^+^ entry ratio during an AP all show slight changes as *g*_c_ increases. Similar to parameter *p*, there is no obvious relationship between Q_overlap_ and Na^+^ entry ratio (Figures 4C,D). Thus, the modulations of AP efficiency with coupling conductance *g*_c_ is not through altering the overlap load between Na^+^ and K^+^ currents during the repolarizing phase of APs. It is worth noting that the passive dendrite with large values of *p* is so small that increasing *g*_c_ has little effects on somatic APs and their underlying currents. Then, four items for relevant APs show little changes with coupling conductance (Figure 4, *p* = 0.8).

**Figure 5:**
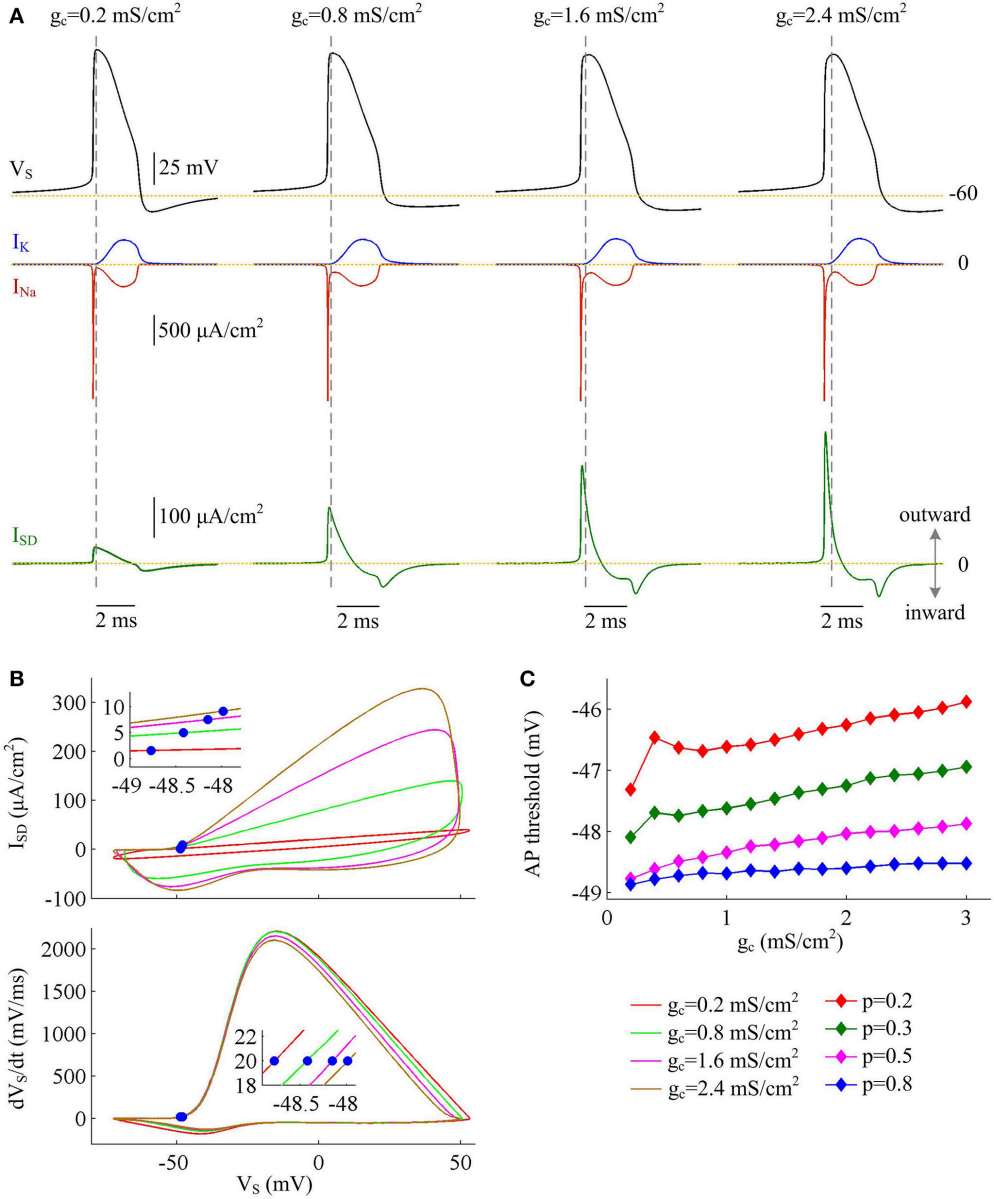
Inefficient APs arise from increased overlap between *I*_SD_ and *I*_Na_ as coupling conductance increases. **(A)** *I*_Na_ (red), *I*_K_ (blue), and *I*_SD_ (green) underlying the APs with different values of coupling conductance, which have been indicated on the top of the panels. Morphological parameter is *p* = 0.5. **(B)** Top panel depicts *I*_SD_-*V*_S_ curve for each AP shown in **(A)**. Bottom panel is the phase plot of d*V*_S_/d*t* vs. *V*_S_ for corresponding AP. Blue dots indicate where APs are initiated. **(C)** AP threshold is plotted as a function of *g*_c_ with different values of *p*. Dendritic input is $I_{\text{D}}=2\;\mu \text{A}/\text{c}{\text{m}}^{\text{2}}$.

The simulations of varying dendrite area may lead one to hypothesize that it is the increase of outward current *I*_SD_ that is the primary effect in the increase of Na^+^ entry ratio with each AP at higher coupling conductance. To test this hypothesis, we depict *I*_Na_, *I*_K_, and *I*_SD_ associated with the APs. It is shown that *I*_Na_ and *I*_K_ both change slightly as coupling conductance *g*_c_ varies (Figure 5A, center). However, internal current *I*_SD_ becomes progressively more prominent with *g*_c_ (Figure 5A, bottom), especially during the rising phase of the spike. The presence of such inhibitory current at the subthreshold potentials antagonizes inward Na^+^ current and makes it become self-sustaining at a more depolarized voltage (Figure 5B), which results in a higher AP threshold (Figure 5C). Combined with falling AP peak, the minimal Na^+^ load needed to produce the upstroke of AP decreases with *g*_c_. Further, the presence of outward *I*_SD_ during the depolarizing phase of AP leads to the overlap between with Na^+^ influx. Under this condition, model I neuron has to import more Na^+^ ions to compete with *I*_SD_ and generate the fast upstroke of APs. Then, the total Na^+^ load during an AP is increased and corresponding Na^+^ entry ratio gets larger. Therefore, the increase in excess Na^+^ entry induced by increasing coupling conductance is largely owing to the increased overlap between outward *I*_SD_ and Na^+^ influx during the depolarizing phase of the AP.

### Activating inward Ca^2+^ current in dendrites increases AP efficiency

**Figure 6:**
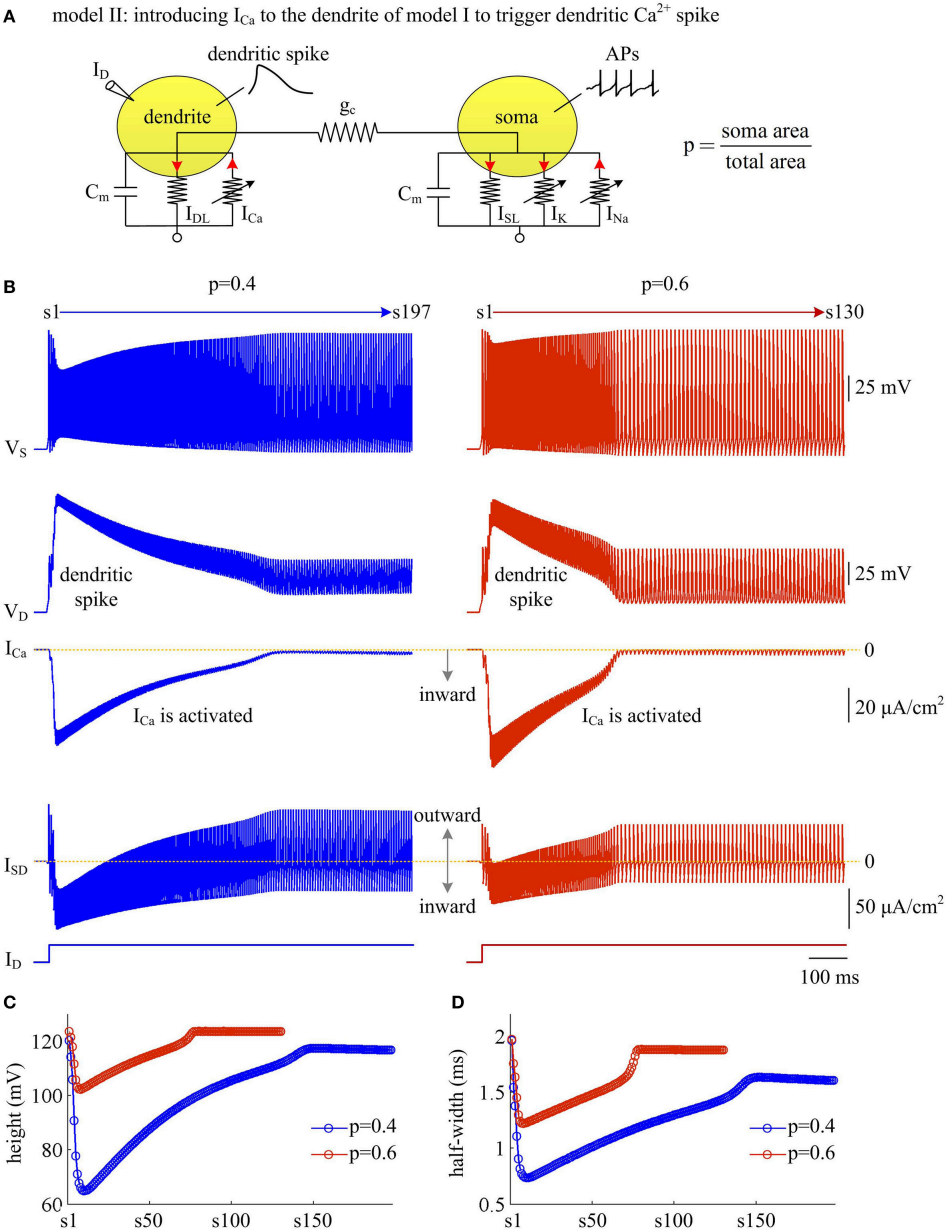
Activating Ca^2+^ current in dendritic chamber results in dendritic spike in model II. **(A)** Schematic of model II. An inward Ca^2+^ current *I*_Ca_ is introduced to the dendritic chamber of model I. **(B)** Sample responses recorded in the soma with *p* = 0.4 and 0.6. Somatic voltage *V*_S_, dendritic voltage *V*_D_, Ca^2+^ current *I*_Ca_ and internal current *I*_SD_ are plotted against time. With *p* = 0.4 (left), there are 197 APs generated during the interval of 1,000 ms, and we number them s1 through s197 from left to right. With *p* = 0.6 (right), there are 130 APs during the interval of 1,000 ms, and we number them s1 through s130 from left to right. **(C)** The height of each AP with *p* = 0.4 and 0.6. **(D)** The half-width of each AP with *p* = 0.4 and 0.6. Note that both height and half-width show marked decrease during the course of dendritic Ca^2+^ spike. $I_{\text{D}}=5\;\mu \text{A}/\text{c}{\text{m}}^{\text{2}}$ and $g_{\text{c}}=0.3\;\text{mS}/\text{c}{\text{m}}^{\text{2}}$.

With a simple two-compartment model, we have simulated how the passive properties of the dendrite modulate the energy efficiency of APs. Our next step is to examine the effects of active currents in dendrites. To achieve this goal, we introduce a voltage-dependent Ca^2+^ current *I*_Ca_ to the passive dendrite of model I and create model II (Figure 6A). Constant input *I*_D_ is applied to activate slow *I*_Ca_ and trigger APs. Activating active Ca^2+^ channel results in a regenerative response in dendritic chamber (Figure 6B), which is referred to as dendritic Ca^2+^ spike. Such all-or-none event in dendritic chamber leads the neuron to generate a burst of high-frequency APs at the onset of input *I*_D_. In this case, the spike train recorded in somatic chamber is no longer periodic (Figure 6B, top). Meanwhile the AP shape also varies as *I*_Ca_ is activated. In particular, the height and half-width of the AP both decrease at first and then increase during the course of dendritic spike (Figures 6C,D). Increasing dendrite area extends Ca^2+^ spike and increases the intensity of internal current *I*_SD_, thus effectively enhancing the modulations of somatic APs.

**Figure 7:**
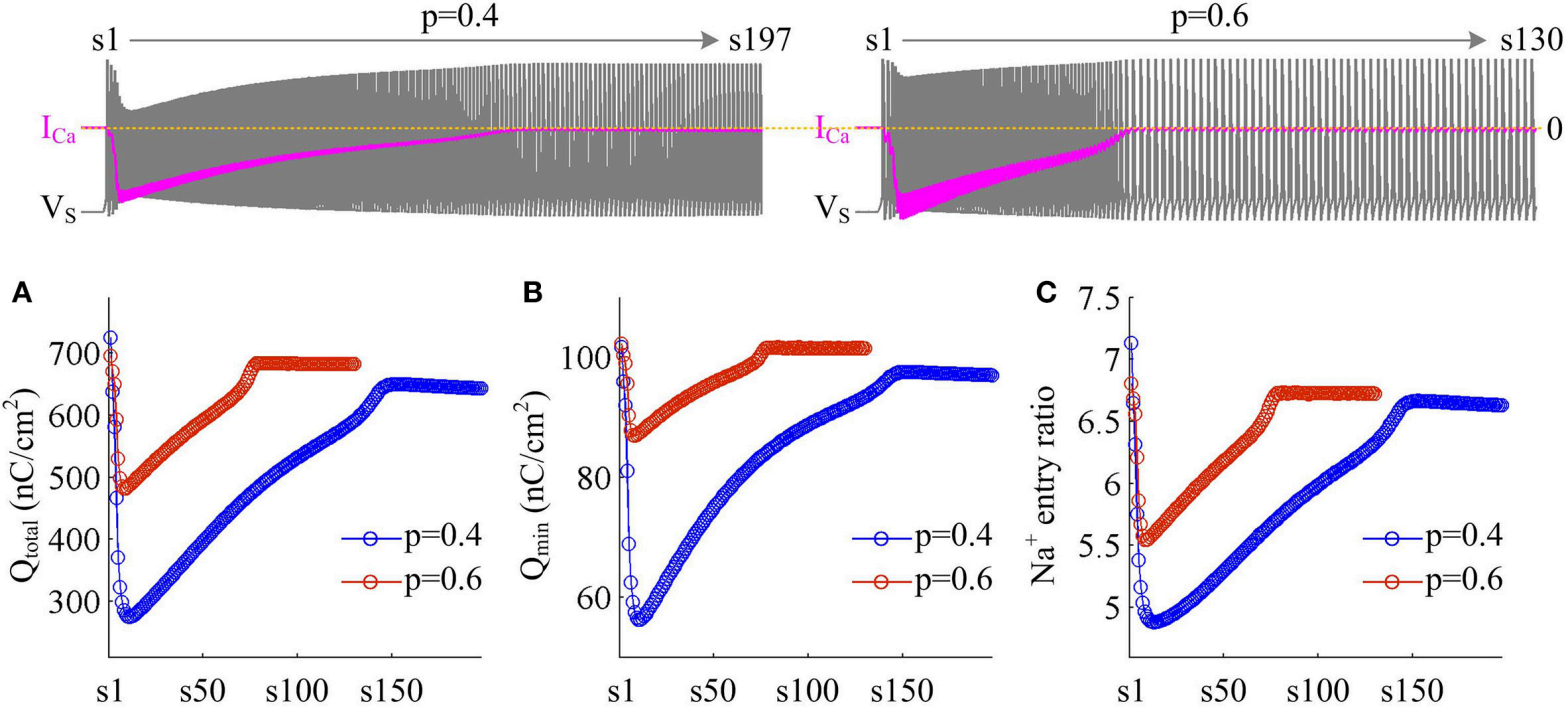
Dendritic Ca^2+^ spike increases metabolic efficiency of APs. **(A)** Total Na^+^ load Q_total_, **(B)** minimal Na^+^ load Q_min_, and **(C)** Na^+^ entry ratio are respectively computed for each AP during the activation of *I*_Ca_ with *p* = 0.4 and 0.6. Top panels show the response of *V*_S_ (gray) and *I*_Ca_ (pink) recorded in the model II with each value of *p*. $I_{\text{D}}=5\;\mu \text{A}/\text{c}{\text{m}}^{\text{2}}$ and $g_{\text{c}}=0.3\;\text{mS}/\text{c}{\text{m}}^{\text{2}}$.

We use Na^+^ entry ratio to quantify the metabolic efficiency of the APs associated with dendritic Ca^2+^ spike. It is found that the total Na^+^ load (Figure 7A) and the minimal Na^+^ load (Figure 7B) during each AP show similar trends as *I*_Ca_ is activated. Two items both decay down before *I*_Ca_ reaches its peak value and then increase in the second phase of dendritic spike. That is, the metabolic cost per spike is significantly reduced by the activation of inward *I*_Ca_. Interestingly, Na^+^ entry ratio also first quickly decreases to a minimal value and then slowly rises to a lower plateau level (Figure 7C). It is indicated that dendritic Ca^2+^ spike makes APs become more efficient to use Na^+^ influx to generate their depolarization, thus increasing their metabolic efficiency.

**Figure 8:**
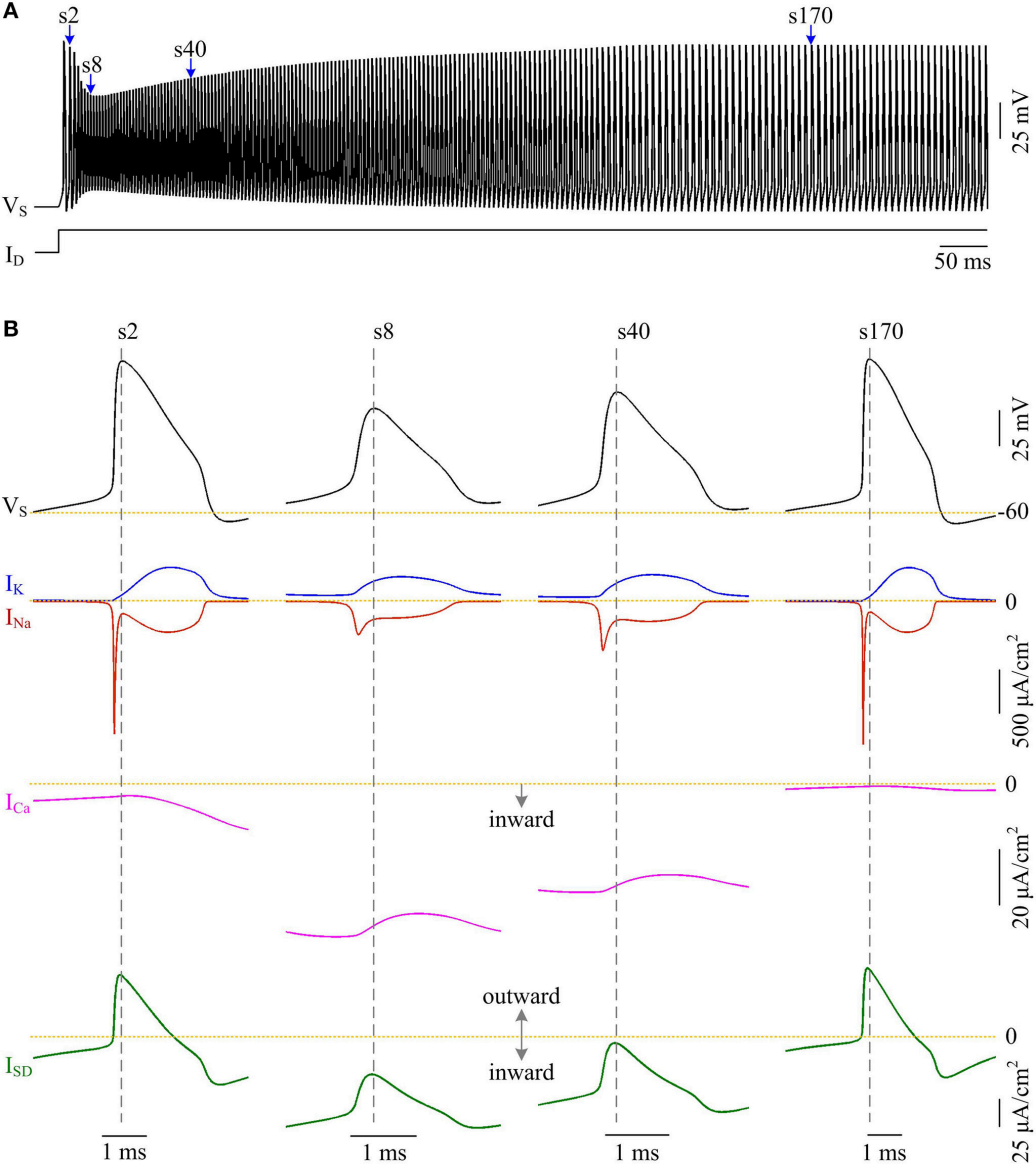
Activating *I*_Ca_ makes the overlap between *I*_SD_ and *I*_Na_ disappear and increases AP efficiency. **(A)** Spike train recorded in the model II neuron with *p* = 0.4. We extract four APs indicated by blue arrows to examine their underlying currents. **(B)** *I*_Na_ (red), *I*_K_ (blue), *I*_Ca_ (pink) and *I*_SD_ (green) underlying the 2nd, 8th, 40th, and 170th APs. $I_{\text{D}}=5\;\mu \text{A}/\text{c}{\text{m}}^{\text{2}}$ and $g_{\text{c}}=0.3\;\text{mS}/\text{c}{\text{m}}^{\text{2}}$.

Plots of *I*_Na_, *I*_K_, *I*_SD_, and *I*_Ca_ underlying individual spikes reveal that the efficient APs triggered by dendritic Ca^2+^ spike are also owing to the modulation of internal current *I*_SD_ (Figure 8). Specifically, once *V*_D_ is forced to reach a threshold voltage, slow inward *I*_Ca_ is activated and then a broader Ca^2+^ spike is initiated in dendritic chamber. Such regenerative event at the slow timescale results in a prolonged local depolarization of *V*_D_, which effectively decreases the outward level of *I*_SD_ and even switches its direction from outward to inward (Figures 6B, 8B). When *I*_SD_ still flows out of the soma, such as in the last phase of dendritic spike, decreasing its intensity reduces its overlap with Na^+^ influx, thus increasing AP efficiency. When *I*_SD_ flows into the soma, the overlap between it and Na^+^ influx during the upstroke of the AP disappears. Instead, the inward *I*_SD_ cooperates with Na^+^ influx to contribute to the depolarization of somatic membrane, thus effectively increasing the efficiency of Na^+^ entry. Meanwhile, the depolarizing *I*_SD_ induced by the activation of *I*_Ca_ also significantly reduces the intensity of *I*_Na_ and *I*_K_, especially the former (Figure 8B, center). Under this condition, the height of relevant AP is reduced (Figure 6C) and its half-width gets narrow (Figure 6D). Then, the total Na^+^ load per spike is significantly reduced during dendritic Ca^2+^ spike. As a result, activating *I*_Ca_ in the dendrite of model II neuron reduces the energy cost of somatic APs and makes them metabolically efficient.

### Activating outward Ca^2+^-activated K^+^ current in dendrites decreases AP efficiency

**Figure 9:**
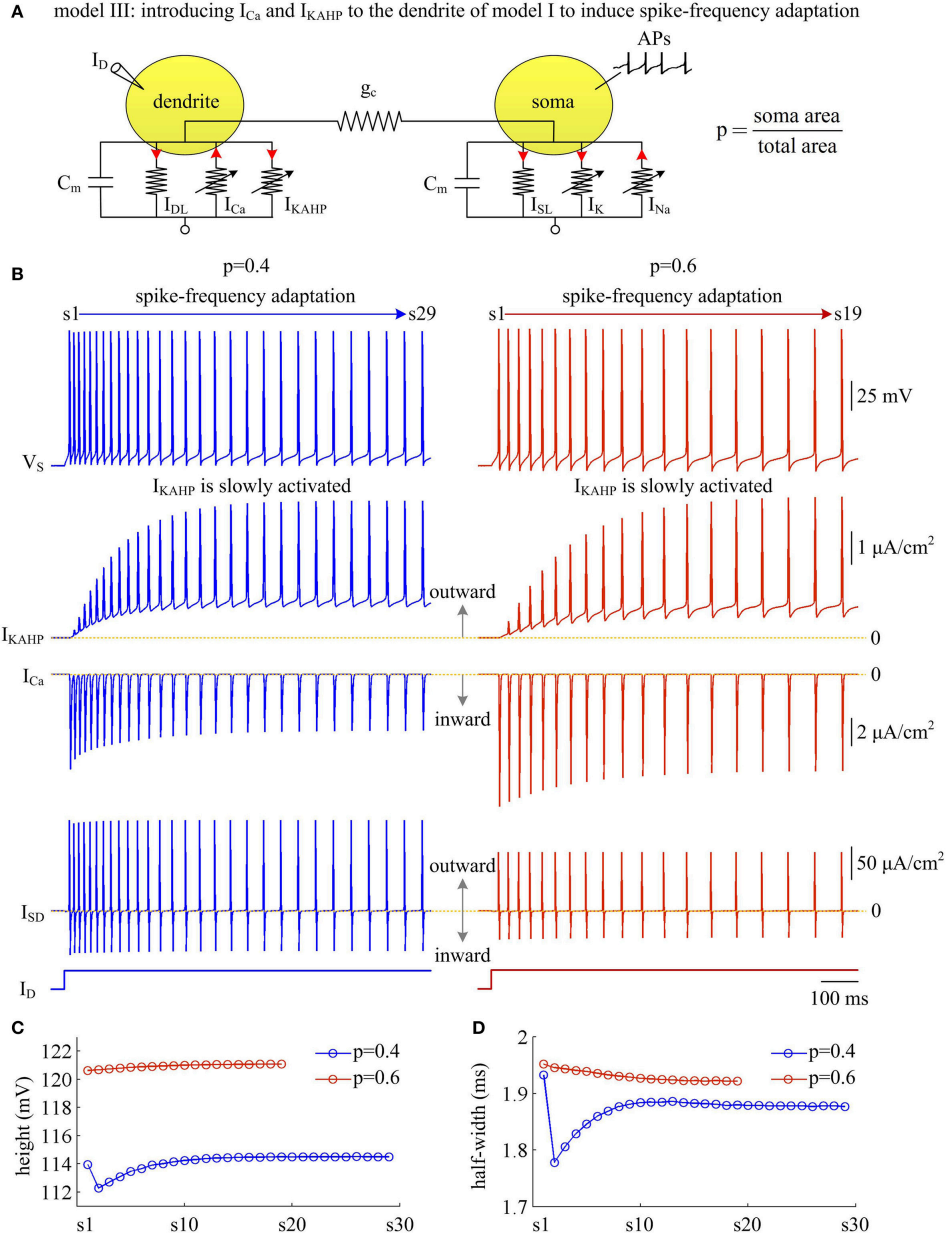
Activating *I*_KAHP_ in dendritic chamber results in SFA in model III. **(A)** Schematic of model III. An inward Ca^2+^ current *I*_Ca_ and a Ca^2+^-activated outward K^+^ current *I*_KAHP_ are introduced to the dendritic chamber of model I. **(B)** Sample responses recorded in the soma with *p* = 0.4 and 0.6. *V*_S_, *I*_KAHP_, *I*_Ca_, and *I*_SD_ are plotted against time. With *p* = 0.4 (left), there are 29 APs generated during the interval of 1,000 ms, and we number them s1 through s29 from left to right. With *p* = 0.6 (right), there are 19 APs during the interval of 1,000 ms, and we number them s1 through s19 from left to right. As *I*_KAHP_ is activated, the firing rate decays down to a lower steady-state level, and model III neuron generates SFA. **(C)** The height of each AP with *p* = 0.4 and 0.6. **(D)** The half-width of each AP with *p* = 0.4 and 0.6. $I_{\text{D}}=2\;\mu \text{A}/\text{c}{\text{m}}^{\text{2}}$ and $g_{\text{c}}=0.6\;\text{mS}/\text{c}{\text{m}}^{\text{2}}$.

Apart from inward active currents, there are also active currents flowing out of the dendrites, which mainly hyperpolarize dendritic membrane voltage. To determine how these inhibitory currents affect AP efficiency, we introduce a Ca^2+^-activated K^+^ current *I*_KAHP_ into the dendritic chamber of model II and create model III (Figure 9A). The activation of *I*_KAHP_ occurs at a slower timescale than the fast dynamics of APs, which includes a form of negative feedback to cell excitability. Here, we examine the Na^+^ entry efficiency of the simulated APs as *I*_KAHP_ is activated.

Similar to above simulations, a constant input *I*_D_ is applied to activate *I*_KAHP_ and evoke APs. We find that the activation of *I*_KAHP_ in dendritic chamber reduces the firing rate to a lower steady-state level (Figure 9B, top), i.e., SFA occurs. During the course of SFA, *I*_KAHP_ increases with co-occurring APs (Figure 9B, center). Once it is sufficiently activated, the firing rate no longer decreases and reaches a steady state. Increasing dendrite area produces little effects on the peak of *I*_KAHP_, whereas it increases the intensity of this current at the subthreshold voltages. Such manipulation also increases the amplitude of internal current *I*_SD_. In this case, activating *I*_KAHP_ with small dendritic chamber has less effect on the shape of somatic APs (Figures 9C,D). Unlike *I*_SD_, increasing dendrite area reduces the intensity of inward *I*_Ca_, which competes with outward *I*_KAHP_ in dendritic chamber to result in distinct modulations of AP shape. In particular, the AP half-width shows opposite evolutions with the activation of *I*_KAHP_ at different values of *p* (Figure 9D). Further, there is a marked decrease in AP shape between the first two APs with *p* = 0.4. This is because outward *I*_KAHP_ is close to 0 μA/cm^2^ during the first AP, which begins to take effects in the second AP. In contrast, inward *I*_Ca_ is relatively strong in the first AP, and gradually decays as *I*_KAHP_ activates. The non-linear competition between *I*_KAHP_ and *I*_Ca_ leads to the marked decrease in AP height and half-width.

**Figure 10:**
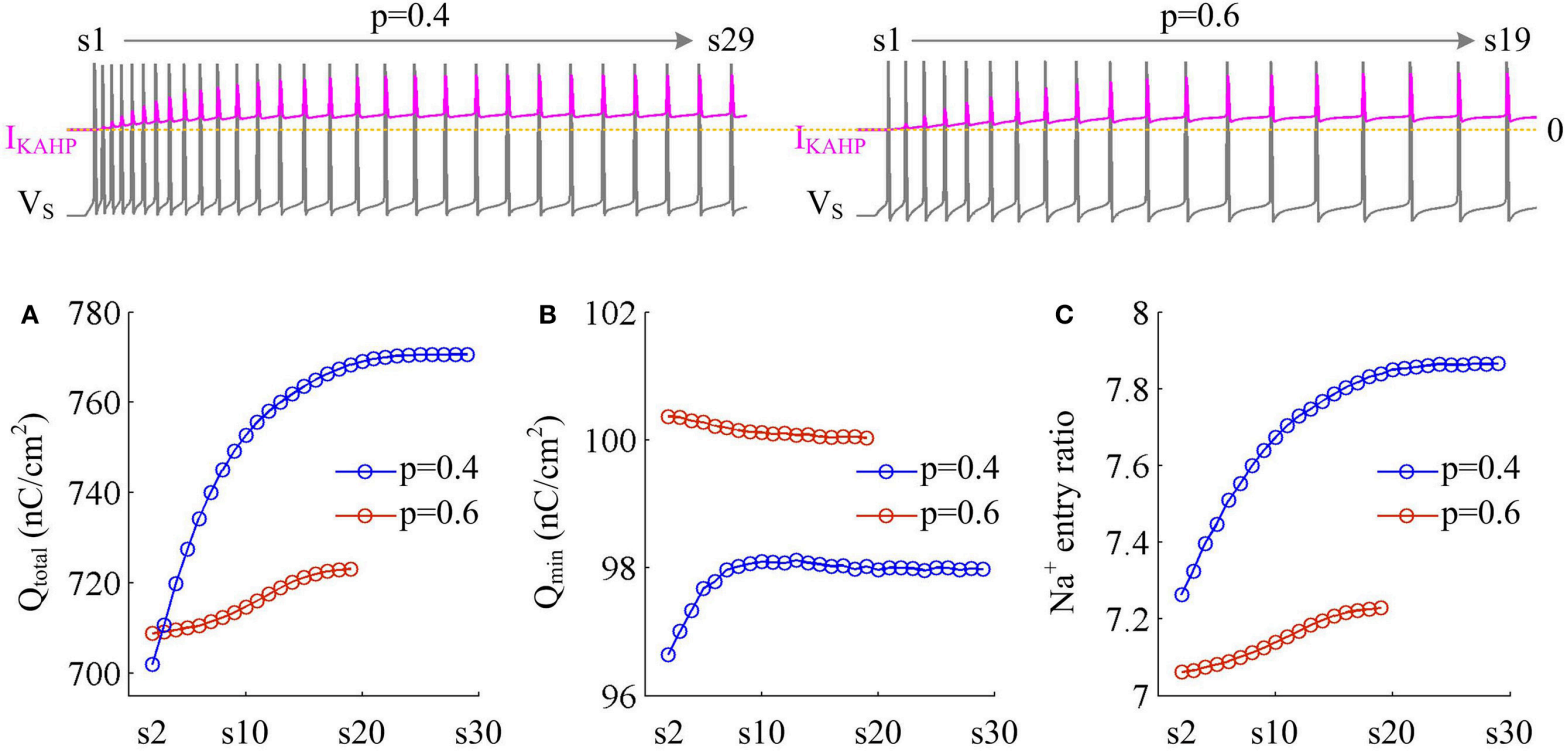
Metabolic efficiency of the AP is reduced as *I*_KAHP_ is activated. **(A)** Total Na^+^ load Q_total_, **(B)** minimal Na^+^ load Q_min_, and **(C)** Na^+^ entry ratio are respectively calculated for each AP during the activation of *I*_KAHP_ with *p* = 0.4 and 0.6. Top panels show the response of *V*_S_ (gray) and *I*_KAHP_ (pink) recorded in the model III with each value of *p*. Note that our examination starts from the 2nd AP, since *I*_KAHP_ is totally unactivated during the 1st AP. $I_{\text{D}}=2\;\mu \text{A}/\text{c}{\text{m}}^{\text{2}}$ and $g_{\text{c}}=0.6\;\text{mS}/\text{c}{\text{m}}^{\text{2}}$.

We measure the Na^+^ entry ratio per AP during the time course of SFA. The results show that activating *I*_KAHP_ in dendritic chamber increases the total Na^+^ load per spike (Figure 10A), thus increasing the metabolic cost of relevant AP. The minimal Na^+^ load (Figure 10B) with different values of *p* show opposite trends as *I*_Ca_ is activated. This arises from the interactions of *I*_KAHP_ with different intensities of *I*_Ca_ induced by changing dendrite area. By calculating Q_total_/Q_min_ for each AP, we show that the Na^+^ entry ratio increases as firing rate is reduced (Figure 10C). This indicates that activating outward *I*_KAHP_ in the dendrite makes APs become inefficient to use Na^+^ entry to generate their depolarization, thus reducing their metabolic efficiency.

**Figure 11:**
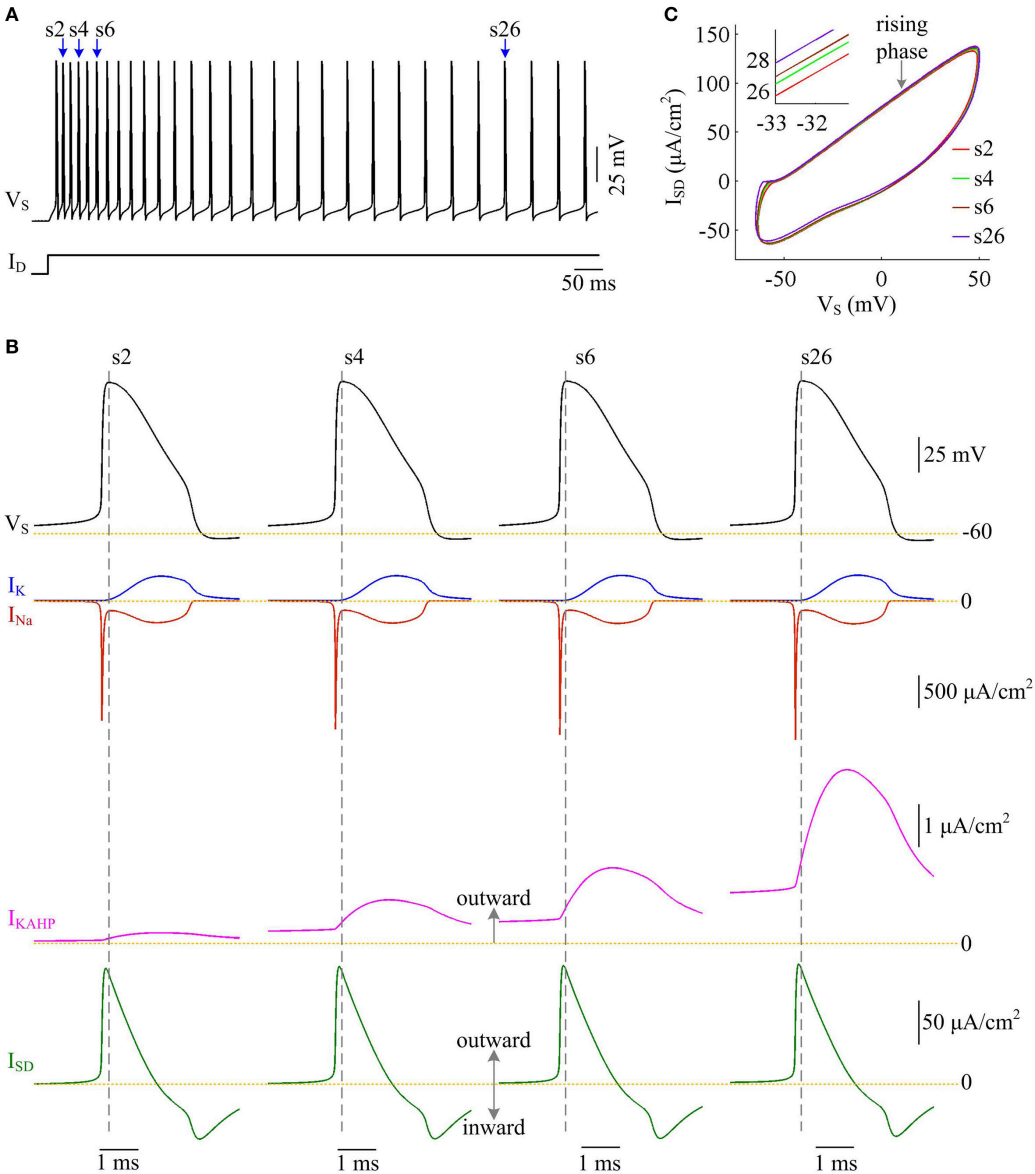
Activating *I*_KAHP_ increases the overlap between *I*_SD_ and *I*_Na_ and reduces AP efficiency. **(A)** Spike train recorded in the model III neuron with *p* = 0.4. Four APs indicated by blue arrows are extracted to examine their underlying currents. **(B)** *I*_Na_ (red), *I*_K_ (blue), *I*_KAHP_ (pink) and *I*_SD_ (green) underlying the 2nd, 4th, 6th, and 26th APs. **(C)** *I*_SD_-*V*_S_ curves for four APs. Activating *I*_KAHP_ in dendritic chamber increases the outward level of *I*_SD_ during the rising phase of the APs (see inset), which overlaps with *I*_Na_ and reduces efficiency of Na^+^ entry. $I_{\text{D}}=2\;\mu \text{A}/\text{c}{\text{m}}^{\text{2}}$ and $g_{\text{c}}=0.6\;\text{mS}/\text{c}{\text{m}}^{\text{2}}$.

We examine the ionic currents underlying the recorded spike trains with *p* = 0.4 (Figure 11A). We find that the intensity of outward *I*_KAHP_ during each AP is much lower compared to *I*_Na_, *I*_K_, or *I*_SD_, even when it is sufficiently activated (Figure 11B). But activating such inhibitory current in dendritic chamber facilitates the hyperpolarization of its membrane voltage and results in slight increase in the outward level of internal current *I*_SD_ (Figures 11B,C). Then, the overlap between outward *I*_SD_ and Na^+^ influx during an AP becomes progressively more prominent as *I*_KAHP_ is activated. The augment in their temporal overlap leads corresponding AP to import more Na^+^ ions for achieving somatic depolarization (Figure 10A), which adds an additional metabolic cost. In this case, more of Na^+^ influx is employed to compete with outward *I*_SD_, which effectively increases the excess Na^+^ entry ratio (Figure 10C). Thus, activating outward *I*_KAHP_ in the dendrites reduces the efficient use of Na^+^ entry and makes APs metabolically inefficient.

**Figure 12:**
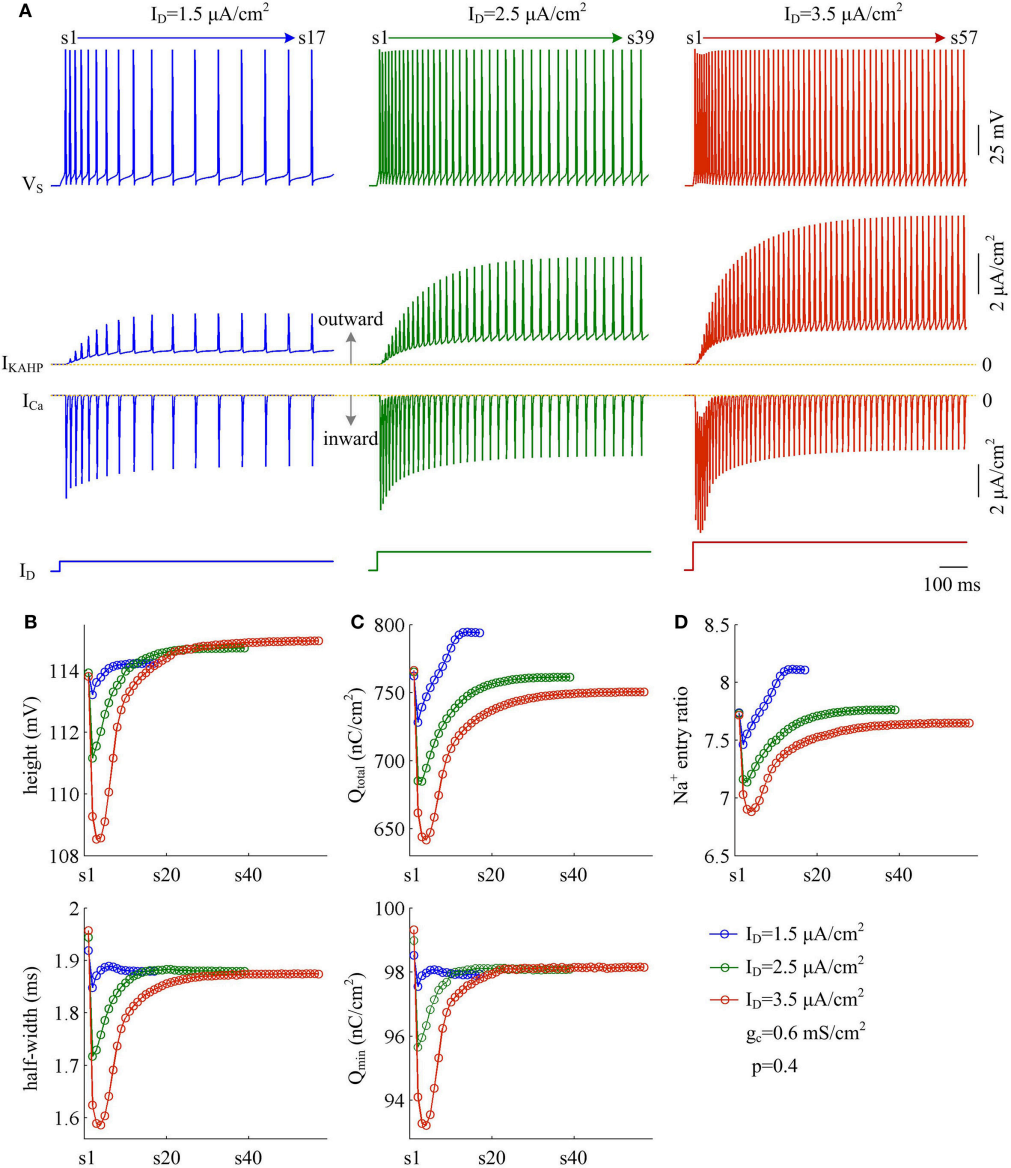
Activation of *I*_KAHP_ by strong dendritic input results in inefficient APs. **(A)** Sample responses of the model III neuron evoked by $I_{\text{D}}=1.5\;\mu \text{A}/\text{c}{\text{m}}^{\text{2}}$, 2.5 μA/cm^2^, and 3.5 μA/cm^2^. *V*_S_, *I*_KAHP_, and *I*_Ca_ are plotted against time. During the interval of 1000 ms, model III neuron respectively generates 17, 39, and 57 APs. **(B)** The height (top) and half-width (bottom) of each AP with three values of *I*_D_. **(C)** Total Na^+^ load Q_total_ (top) and minimal Na^+^ load Q_min_ (bottom) of each AP. **(D)** Excess Na^+^ entry ratio of each AP. Note that three intensities of *I*_D_ are all below the threshold for activating *I*_Ca_ to trigger dendritic Ca^2+^ spike. Once *I*_D_ reaches that threshold, the activated *I*_Ca_ dominates the outputs of model III neuron, and the SFA will disappear. *p* = 0.4 and $g_{\text{c}}=0.6\;\text{mS}/\text{c}{\text{m}}^{\text{2}}$.

Moreover, the effects of adaptation currents on APs have been shown to be dependent on current stimulus (Prescott et al., 2006; Prescott and Sejnowski, 2008; Benda et al., 2010; Yi et al., 2015b). Here we use model III neuron to simulate how the metabolic efficiency of APs depends on the activation of *I*_KAHP_ as dendritic input is varied. We measure height and half-width, total and minimal Na^+^ load, and Na^+^ entry ratio for simulated APs. It is found that increasing dendritic input makes *I*_KAHP_ stronger and extends its activation procedure (Figure 12A, center). Then, activating outward *I*_KAHP_ with strong *I*_D_ produces larger effects on AP height and half-width (Figure 12B), total Na^+^ load and minimal Na^+^ load per spike (Figure 12C), as well as excess Na^+^ entry ratio (Figure 12D). Even so, these items show similar trends with the activation of *I*_KAHP_. In particular, our predictions are reproducible in the cases of different dendritic inputs. That is, activating inhibitory *I*_KAHP_ in dendritic chamber increases both energy cost (Figure 12C, top) and Na^+^ entry ratio (Figure 12D) per spike, which makes relevant AP metabolically inefficient. Note that applying strong *I*_D_ to dendritic chamber simultaneously increases the intensity of inward *I*_Ca_, especially during the initial APs after the onset of injection. Since outward *I*_KAHP_ is relatively weak in this phase, *I*_Ca_ dominates the outcome of their competition, which results in the marked decrease in AP shape, total Na^+^ load, minimal Na^+^ load and Na^+^ entry ratio. After that, *I*_Ca_ decays and then the activated *I*_KAHP_ dominates the outcome of their competition, which results in SFA. Thus, we neglect the decrease in each item when we examine the effects of activating *I*_KAHP_ on AP efficiency with different stimulus.

## Discussion

Our simulations develop three two-compartment biophysical models to describe the intrinsic properties of the dendrites and reproduce the APs initiated in cortical pyramidal cells. The relationships between the dendritic properties, the energy efficiency of APs, and the currents underlying relevant AP are determined. The excess Na^+^ entry ratio is applied to quantify the efficiency with which Na^+^ influx is used for AP formation. These calculations allow us to identify how passive and active properties of the dendrites modulate AP efficiency in each model.

Our approach is to forward engineer simple point-neuron models to better understand how the passive and active dendrites affect the energy efficiency of somatic/axonal APs. We create the models only as complicated as required to reproduce the phenomena of interest. Note that the morphology and biophysics of real dendrites is extremely complicated and particular to specific cells. Creating a high-dimensional biophysical model to capture these non-linearities is reasonably straightforward. However, it may fail to provide a deeper insight than the physiological experiments upon which is based. In our forward engineered models, we exclude the extraneous details of pyramidal cells and reduce the number of core parameters to workable proportions. Such reduced point-neuron models allow us to simulate the core process of interest and gain a greater understanding of fundamental principles than biophysically realistic, extensive models.

We first examine the effects of two passive properties with our biophysical models. One relevant parameter is the ratio of dendrite area to total membrane area, and the other one is the internal coupling conductance connecting chambers. By systematically varying them within the model, we find that increasing dendrite area or coupling conductance both result in a marked increase in the internal current flowing between two chambers. This is an outward current flowing out of the soma, which overlaps with Na^+^ influx during the upstroke of the AP. Increasing the intensity of such inhibitory current makes the overlap more significant. Then, the Na^+^ entry efficiency of relevant AP is reduced. It has been shown that the voltage-gated channels or pumps must fit into a limited membrane area (Faisal et al., 2005). With single-compartment models, Sengupta et al. (2013) demonstrate that membrane area constrains the available channels used for the information coding, the signaling rate and the energy efficiency of the cell. By simulating two-compartment models, we predict that the membrane area of either soma or dendrite is a determinant of AP efficiency, which is comparable to their study.

We also simulate the metabolic efficiency of APs associated with active dendrites. Two types of dendritic channels are examined. One is inward Ca^2+^ current *I*_Ca_, and the other one is outward Ca^2+^-dependent K^+^ current *I*_KAHP_. Their activations both occur at slower timescales than the fast dynamics of spike initiation, which allows us to observe how they modulate AP efficiency in a recorded spike train. Our simulations show that activating active current in dendrites can either enhance or reduce the excess Na^+^ entry ratio of somatic AP, depending on whether it is depolarizing (inward) or hyperpolarizing (outward). The activation of inward *I*_Ca_ results in a local depolarization in dendritic chamber and evokes dendritic spike. Such event effectively decreases the outward level of internal current *I*_SD_ and controls it to flow into the soma. In this case, internal current *I*_SD_ is not to overlap and compete with Na^+^ influx but to cooperate with it to depolarize somatic membrane, thus increasing AP efficiency. On the contrary, activating *I*_KAHP_ hyperpolarizes dendritic membrane and increases the outward level of *I*_SD_. Such event results in more overlap of inhibitory *I*_SD_ and Na^+^ influx, thus decreasing AP efficiency.

The existence of inward active currents (including Na^+^, NMDA and Ca^2+^) in dendrites endows them with powerful ability of synaptic integration (Spruston, 2008; Major et al., 2013; Stuart and Spruston, 2015; Tran-Van-Minh et al., 2015). Particularly, the dendritic spike arising from their activation completely alters the relative importance of synaptic inputs (Larkum et al., 2001, 2004), which lies at the heart of cortical computation (Major et al., 2013; Stuart and Spruston, 2015; Tran-Van-Minh et al., 2015). *In vitro* experiments have observed that dendritic Ca^2+^ spike triggers a burst of APs in the soma/axon and switches the firing mode of the cell to bursting (Williams and Stuart, 1999; Larkum and Zhu, 2002; Larkum et al., 2004; Palmer et al., 2012; Larkum, 2013). With a two-compartment model, our earlier study (Yi et al., 2017) has successfully reproduced this observation and explained how dendritic spike participates in somatic AP initiation. In present study, we show that the activation of Ca^2+^ current in apical dendrites makes Na^+^ entry become efficiently used by APs for their depolarization, thus facilitating the effective utilization of metabolic energy. Our simulations suggest that the supralinear integration operated by dendritic Ca^2+^ spike is a contributory factor for the metabolically efficient coding by cortical pyramidal cells. Note that we only consider slow Ca^2+^ current in our simulations. The effects of other active channels, such as NMDA or Na^+^, need to be examined in future work.

Outward *I*_KAHP_ in dendritic sites is an ionic mechanism for causing SFA. In fact, the *I*_KAHP_ in soma/axon can also lead the cell to adapt its spike frequency (Benda et al., 2010). Similarly, other inhibitory currents, including voltage-gated K^+^ current *I*_M_ (Brown and Adams, 1980) and Na^+^-activated K^+^ current *I*_KNa_ (Wang et al., 2003), are also potential mechanisms for inducing SFA on slow timescales. With single-compartment models, our earlier study (Yi et al., 2016a) has showed that activating *I*_M_ or *I*_KAHP_ in soma/axon directly leads to the overlap between with Na^+^ influx during the depolarizing phase of AP, effectively increasing the Na^+^ load to achieve depolarization, and thus resulting in an inefficient AP with higher energy consumption. Our present study finds that the activation of *I*_KAHP_ in dendritic chamber takes effects in a different way. It directly increases the outward level of internal current *I*_SD_, which overlaps with Na^+^ influx in the soma and reduces AP efficiency. Even so, the similar outcomes of their activation indicate that the presence of slow inhibitory currents in dendrites, soma or axon makes an AP less efficiently use Na^+^ entry for its depolarization. Note that SFA is a common strategy used by neurons to encode signals, which is ubiquitous in the central nervous system (Sharpee et al., 2006; Wark et al., 2007). It has been shown to effectively improve neural computation (Benda et al., 2005, 2010; Sharpee et al., 2006; Wark et al., 2007; Peron and Gabbiani, 2009). In this sense, the ionic currents inducing SFA should be contributory factors for enhancing the metabolic efficiency of information coding. Their relationship needs further investigation in following works.

Two-compartment model is the minimal neuronal unit for capturing the interaction between dendrites and soma/axon, which has been widely used to describe the input-output transfer of single pyramidal cell. In particular, our earlier studies (Yi et al., 2014a,b, 2015b, 2016b, 2017) have demonstrated that dendrite area, dendritic spike and coupling conductance all play crucial roles in determining the transfer function of two-compartment model neurons. For example, they could alter the dynamic basis of spike initiation (Yi et al., 2014a, 2017), the outcome of subthreshold competition between opposite currents (Yi et al., 2014a,b, 2015b), the dynamics of AP threshold (Yi et al., 2016b), the electric field threshold for triggering spikes (Yi et al., 2014b), and the field-induced SFA (Yi et al., 2015b). In current study, we find that the effects of them on internal current flowing between chambers are also translated into distinct modulation of AP efficiency. These simulations demonstrate that such kind of point-neuron models is an effective tool for predicting how passive and active dendrites participate in simple neural computation. Their predictive power will hopefully result in increased applications of these models within relevant fields.

Experimental and computational approaches have been used to determine the energy efficiency of APs. A major determinant of AP efficiency is the overlap of inward and outward currents (Sengupta et al., 2010; Niven, 2016), which is highly variable between neurons. Some contributory factors have been identified, including conductance magnitudes of Na^+^ or K^+^ (Hasenstaub et al., 2010; Sengupta et al., 2010), kinetics of their activation and inactivation (Crotty and Levy, 2007; Hasenstaub et al., 2010; Sengupta et al., 2010; Moujahid and d'Anjou, 2012; Yi et al., 2016a), temperature (Moujahid and d'Anjou, 2012; Yu et al., 2012), membrane capacitances (Crotty and Levy, 2007; Sengupta et al., 2010), AP shape (Carter and Bean, 2009), spike threshold dynamics (Yi et al., 2015a), and cell size (Sengupta et al., 2013). All of them are shown to alter the overlap between inward Na^+^ and outward K^+^ currents to determine AP efficiency. For a specific cell, AP efficiency also varies across different parts (Alle et al., 2009; Schmidt-Hieber and Bischofberger, 2010). Axonal APs exhibit relatively little overlap in Na^+^ and K^+^ currents than soma, resulting in higher efficiency. Such difference is largely owing to the prevalent Kv1.1/1.2 channels (Kole et al., 2007; Shu et al., 2007) and faster Na^+^ kinetics (Schmidt-Hieber and Bischofberger, 2010) in the AIS. Unlike these studies, we predict that the passive properties and active channels in the dendrites cause the variability in AP efficiency by altering the overlap between inward Na^+^ and outward internal current. Thus, the dendrites not only have marked and strong impacts on the final output of neuronal computation, which also affect the energy consumption and efficiency of somatic/axonal APs.

In our simulations, there is significant overlap between inward Na^+^ and outward K^+^ currents during the APs. It arises from the simultaneous activation of two active currents. Under this condition, Na^+^ enters the cell at the same time that K^+^ exits the cell. These fluxes mostly cancel each other, which increase total Na^+^ entry needed to formulate the APs. Such temporal overlap occurs during the repolarization of the AP. Except for delayed rectifier K^+^, *I*_SD_ is another current flowing out of the soma in our two-compartment models. This internal current mainly appears during the upstroke of APs, which effectively leads to unnecessary Na^+^ influx. The passive and active dendrites participate in somatic APs through controlling the *I*_SD_. Calculating the overlap Na^+^ load during the repolarization is unable to measure how outward *I*_SD_ overlaps with Na^+^ influx. Instead, we quantify total Na^+^ entry during the AP relative to the minimal charge necessary for its depolarization. The Na^+^ entry ratio calculated in this way effectively measures how efficiently an AP uses Na^+^ influx to produce its depolarization, which takes the temporal overlap of *I*_SD_ and *I*_Na_ into consideration. In fact, both overlap Na^+^ load and Na^+^ entry ratio are effective measures for determining the potential biophysical causes for the variability in metabolic efficiency of APs among neurons. Based on our simulations, we suggest using Na^+^ entry ratio to measure the AP efficiency when there are outward currents in its depolarizing phase.

Experiments have recorded a myriad of APs with a wide variety of shapes (height and width). An earlier study by Carter and Bean (2009) has suggested that the variability in Na^+^ entry efficiency among neurons primarily arises from different AP shapes rather than Na^+^ channel kinetics. But Sengupta et al. (2010) then showed that AP height and width are poor predictors of energy consumption. Instead, the biophysical properties of ionic channels could be perfectly matched to reduce the overlap between inward and outward currents to minimize the ATP cost of the APs. Similarly, Yu et al. (2012) reported that the changes in AP shape do not explain the modulations of metabolic efficiency of APs with temperature. In our simulations, we find that the passive and active dendrites affect both the shape of APs and the overlap between their opposite currents, thus altering the metabolic cost of somatic APs. Using either Na^+^ entry ratio or AP shape alone is unable to explain the effects of dendritic properties on AP efficiency. This is comparable to the prediction of Sengupta et al. (2010) and Yu et al. (2012). By taking two factors into consideration, our study indicates that the biophysical properties and morphologies of the dendrites are potential causes for altering AP shape and the overlap between inward and outward currents, thus predicting the variable AP efficiency among pyramidal cells.

There are some limitations in our model and technical considerations. First, our simulations only examine the effects of varying one core parameter. Future work should focus on how their possible combinations affect and maximize AP efficiency in pyramidal cells. Second, the morphology and active channels in the dendrites are very complicated for a real cell. Including them in the biophysically-based models to describe their relationships with AP efficiency will surely facilitate our interpretation of the energy expenditure of neuronal computation. Finally, the present study only focuses on the energy efficiency of single AP and not formally simulates the information coding by relevant model neurons. How passive and active dendrites participate in information transmission and then affect its energy efficiency should be examined in following works.

## Conclusion

With biophysical models, we have obtained basic principles about how passive and active dendrites affect the Na^+^ entry efficiency of somatic/axonal APs. Our results emphasize that they are all potential factors for the variability in AP efficiency between pyramidal neurons. By relating dendritic properties to the overlap between Na^+^ influx and internal current, we provide an interpretable insight into their effects. Determining their contributions to the AP efficiency is a first but necessary step toward a mechanistic understanding of how single cell consumes metabolic energy to perform computation. Our models and predictions can be used to examine how other biophysics and morphologies of the dendrites affect spike efficiency. Such examinations are essential for deeply interpreting how these subcellular processes participate in the information processing of neurons and neural circuits.

## Author contributions

Conceived and designed the work: GY, JW, XW, BD. Performed the simulations: GY. Analyzed and interpreted the data: GY, JW. Wrote the paper: GY, JW, XW.

### Conflict of interest statement

The authors declare that the research was conducted in the absence of any commercial or financial relationships that could be construed as a potential conflict of interest.
